# Navigating a newly diagnosed cancer through clinician-facilitated discussions of health-related patient values: a qualitative analysis

**DOI:** 10.1186/s12904-022-00914-7

**Published:** 2022-03-06

**Authors:** Kathleen A. Lynch, Camila Bernal, Danielle R. Romano, Paul Shin, Judith E. Nelson, Molly Okpako, Kelley Anderson, Elizabeth Cruz, Anjali V. Desai, Virginia M. Klimek, Andrew S. Epstein

**Affiliations:** 1 Memorial Sloan Kettering Cancer Center, 641 Lexington Avenue, 7th Floor, New York, NY 10022 USA; 2 Weill Cornell Medical College, New York, NY USA

**Keywords:** Advance care planning, cancer, Communication, Oncology, Values, Patient participation, Nursing, Qualitative

## Abstract

**Background:**

Advance care planning, the process through which patient values and goals are explored and documented, is a core quality indicator in cancer care. However, patient values are predominantly elicited at the end of life; patient values earlier in serious illness are not clearly delineated. The objective of this analysis is to assess the content of patient-verified summaries of health-related values among newly diagnosed cancer outpatients in order to develop a theoretical framework to guide future values discussions and optimize person-centered oncologic care.

**Methods:**

Values summaries among patients with gastrointestinal (GI) cancers or myelodysplastic syndrome (MDS) were extracted from the medical record. Modified grounded theory analysis included interdisciplinary team coding of values summaries to identify key domains; code categorization; and identification of thematic constructs during successive consensus meetings. A final round of coding stratified themes by disease type.

**Results:**

Analysis of 128 patient values summary documents from 67 patients (gastrointestinal [GI] cancers, *n* = 49; myelodysplastic syndrome [MDS], *n* = 18) generated 115 codes across 12 categories. Resultant themes demonstrated patients’ focus on retaining agency, personhood and interpersonal connection amidst practical and existential disruption caused by cancer. Themes coalesced into a theoretical framework with 5 sequenced constructs beginning with the cancer diagnosis, leading to 3 nesting constructs of individual identity (character), interpersonal (communication) preferences and needs, and social identity (connection), signifying sources of meaning and fulfillment. Values differences between GI cancer and MDS patients—including greater focus on normalcy, prognosis, and maintaining professional life among GI patients—reflected the distinct therapeutic options and prognoses across these disease groups.

**Conclusions:**

Patient values reflect goals of meaning-making and fulfillment through individual agency and interpersonal supports in the setting of a newly diagnosed cancer. Early, nurse-led values discussions provide important and patient-specific data that are informative and likely actionable by clinicians in the delivery of person-centered care. Values can also facilitate discussions between patients and families and clarify patient preferences.

**Supplementary Information:**

The online version contains supplementary material available at 10.1186/s12904-022-00914-7.

## Background

A person’s health-related values, (i.e., what is most important to the individual, including hopes, concerns, goals, and preferences) are the foundation for person-centered care, including in oncology where care plans need to align with individuals’ goals and preferences in unique and complicated situations. Cancer interrupts patients’ lives, creates uncertainty about the future, threatens health and life, and disrupts the role patients play in their family and community [1]. Patients want to communicate their values to health care teams, and to receive care that is aligned with what matters most to them [2].

The values of patients with serious illness are often elicited only at the end of life, if at all [3]. However, values should be discussed early on in cancer, regardless of stage or prognosis. Advance care planning, the process through which patient values and goals are explored and documented, is a core quality indicator in cancer care [4]. With increasing recognition of the importance of a holistic [5], *person*-centered approach to care in oncology, studies have begun to assess discussion of patient values early in care [6] not just in advanced [7] or worsening [8] cancer, or at the end of life [9]. We have previously demonstrated the feasibility and acceptability of nurse-led discussions with newly diagnosed cancer patients about their values [6]. We found that normalizing the process of systematically eliciting values early in patients’ outpatient care helped avoid the stigma and apprehension that may be associated with other types of conversations (e.g.*,* goals of care discussions about end-of-life care preferences) for patients, families and clinicians [6].

.While some studies have elucidated what is important to patients at the end of life [7, 9, 10], there remains a dearth of literature on patient values earlier in serious illness. And, despite our recent data showing the acceptability and feasibility of eliciting values of newly-diagnosed cancer patients, the content of such discussions and how they might be used by the clinical team is not yet known. We sought to address these gaps through qualitative analysis of patient-verified summaries documenting nurse-led discussions about health-related values conducted shortly after cancer diagnosis. We aimed to build a theoretical framework of how patients’ values guide their individual approach to confronting the threat of a new cancer diagnosis, communicating with loved ones and healthcare providers, and ultimately making meaning of their illness.

## Methods

This study was performed as a pre-planned component of an institutional quality improvement initiative, the Memorial Sloan Kettering (MSK) “1–2-3 Project,” that has been previously described [11]. Briefly, the 1–2-3 Project involves a structured assessment conducted by the outpatient oncology nurse who works with the patient’s primary oncologist. To ensure reliability, all nurses received communication skills training following the ANP (acknowledgement, normalizing, partnering) framework for empathic communication [6]. Patients are introduced to the values discussion during their second clinic follow-up visit. During this visit, patients receive a one-page “Getting to Know You” handout inviting them to participate in a brief discussion exploring “who you are as a person and what you most value.” This handout includes three example questions for the patient to consider (*What should we know about you as a person to take best care of you? Facing cancer, what gives you strength? What does living well mean to you at this time?*). Patients are encouraged to think about these questions, discuss them with family, make notes, and complete the values discussion with a nurse at their next visit. At the following visit, which averaged 4 weeks later, a nurse uses an evidence-based question guide to elicit patients’ core health-related values. Median discussion length was 15 min (range: 5 min- 30 min). The nurse compiles a one-page summary of the discussion following a structured template (Additional file 1) which is then verified/updated by the patient before it is uploaded to the Electronic Health Record. The patient is given a copy of this summary to share with their loved ones and/or decision-makers. The summaries are revisited quarterly and/or if a patient or clinician wishes to revisit sooner.

The present analysis of values statements was reviewed by the MSK Institutional Review Board (IRB Protocol # × 16–034), who waived informed consent based on minimal risk to participants.

### Data analysis

Nurse summaries of all 1–2-3 Project values discussions conducted between March 2017 (when the values elicitation and documentation phase of the 1–2-3 Project began) and December 2019 were extracted from the medical record and uploaded to Atlas.ti v. 7.5 for analysis. During this time period, values statements were conducted among patients with gastrointestinal (GI) cancers or myelodysplastic syndrome (MDS). GI cancers are a diverse grouping of solid tumors with heterogenous presentations and stages of disease, usually with prognosis measured in months to a small number of years when present in the metastatic setting. In contrast, MDS arises from hematologic abnormalities in the bone marrow, takes many clinical forms, often presents in older individuals, and sometimes develops into acute leukemia. Patients with hematologic and other malignancies underutilize palliative care medicine and face unique barriers to palliative care [12]. Due to their distinct clinical profiles, prognoses, and the dearth of studies on palliative care introduction in hematologic malignancies, GI and MDS were selected as the initial focus for the implementation of 1–2-3 Project.

An interdisciplinary coding team consisting of a physician with post-graduate training in surgical oncology and history of medicine (P.S.), clinical psychologist (D.R.), sociomedical scientist (C.B.), and medical anthropologist (K.A.L.) conducted a modified grounded theory analysis of the values summaries [13]. This modified grounded theory approach, popularized by Charmaz [13], was selected as the most appropriate analytic method because its central goal is to build novel theory from the data by an iterative, inductive process in which the data serve to generate rather than verify hypotheses, while acknowledging and incorporating researcher knowledge of preexisting frameworks into initial annotation and coding, thereby co-producing meaning from reflexive knowledge and the novel, personal narratives of this specific population [14]. Initial codes were organized based on domains of the Values Summary (e.g., “major concerns,” “patient hopes,” “preferences for end-of-life care”) and refined based on open coding of patterns and concepts that emerged from the data. All coders independently reviewed the first ten summaries using the initial codes, noting key points throughout the text, and generating memos for potential new codes. The team met to discuss key points and update the initial coding guide, at which point each coder was randomly assigned ten more summaries. This process was repeated until all summaries were coded. Then, all individual coding files were merged into an Atlas.ti master file and related codes were sorted into categories (axial coding). Axial coding was conducted collaboratively by the coding team. This involved reviewing aggregated statements, or “quote reports,” associated with each code and mapping how the code related to the larger dataset by placing it into a discrete category. In a final round of selective coding, the team reviewed statements within each category to facilitate the identification of major thematic constructs, as well as identify and discuss thematic differences between subgroups (e.g. GI and MDS patients). Study authors met to reach consensus via discussion on the significance of themes and subtopics. A detailed description of each construct with key supporting quotations was summarized in a consensus document. The coders and the senior author (A.S.E.) then used this document as the basis to build a theoretical framework, mapping how each thematic construct related to the rest of the data (e.g. nested, interrelated, or along a pathway). Once consensus was established, the authors visualized this relationship.

## Results

### Study participants and values discussion characteristics

One hundred twenty eight values summaries were completed by 4 oncology nurses for discussions with 67 unique newly diagnosed patients of 3 oncologists [6]. Most (73%) of these patients were diagnosed with GI cancers: of these, 51% had metastatic disease, while 49% had either local or locally-advanced disease. All other patients (27%) had MDS (Table 1). All MDS summaries were completed by a single nurse (K.A.); all GI summaries except two were completed by a second nurse (M.O.).

**Table 1:** Patient Demographics

Baseline Characteristics	GI Cancer (***N*** = 49 Patients)	MDS (***N*** = 18 Patients)
Age (mean age in years, range)	61 (35–87)	68 (34–88)
Gender		
Male	32 (65%)	11 (61%)
Female	17 (35%)	7 (39%)
Malignancy Type **(GI Only)**		
Colorectal (or Small Bowel)	22 (45%)	
Pancreatic	16 (33%)	
Biliary (Gallbladder or Bile Duct)	5 (10%)	
Gastroesophageal	5 (10%)	
Peritoneal	1 (2%)	
Stage **(GI Only)**		
I	1 (2%)	
II	4 (8%)	
III	19 (39%)	
IV	25 (51%)	
Race		
White	38 (78%)	15 (83%)
Asian	6 (12%)	0 (0%)
Black/African American	3 (6%)	2 (11%)
Other	2 (4%)	1 (6%)
Ethnicity		
Hispanic/Latinx	4 (8%)	2 (11%)
Non-Hispanic/Non-Latinx	44 (90%)	15 (83%)
Unknown	1 (2%)	1 (6%)
Marital status		
Married	30 (61%)	11 (61%)
Single	12 (25%)	4 (22%)
Divorced	4 (8%)	2 (11%)
Widowed	3 (6%)	1 (6%)
Religion		
Roman Catholic	17 (35%)	3 (17%)
None	14 (29%)	3 (17%)
Other Christian	11 (22%)	7 (39%)
Jewish	4 (8%)	5 (27%)
Muslim	1 (2%)	0 (0%)
Hindu	1 (2%)	0 (0%)
Unknown	1 (2%)	0 (0%)
Vital Status*		
Alive	23 (47%)	7 (39%)
Deceased	26 (53%)	11 (61%)

*As of 2/19/2021

Fifty nine of the 67 patients completed values discussions over two clinic visits; the full values discussion was completed at a single clinic visit for 8 additional patients, after a protocol amendment to the larger 1–2-3 Project. Some patients completed more than one summary: of the MDS patients (*n* = 18), 50% completed a single values summary; those with more than one summary completed their subsequent discussions within 6 months. Similarly, of the GI patients (*n* = 49) 59% completed a single values summary; those with more than one completed additional summaries 1–7 months after the initial discussion.

### Qualitative results: patients’ navigation of a cancer diagnosis through values discussions

**Fig. 1:**
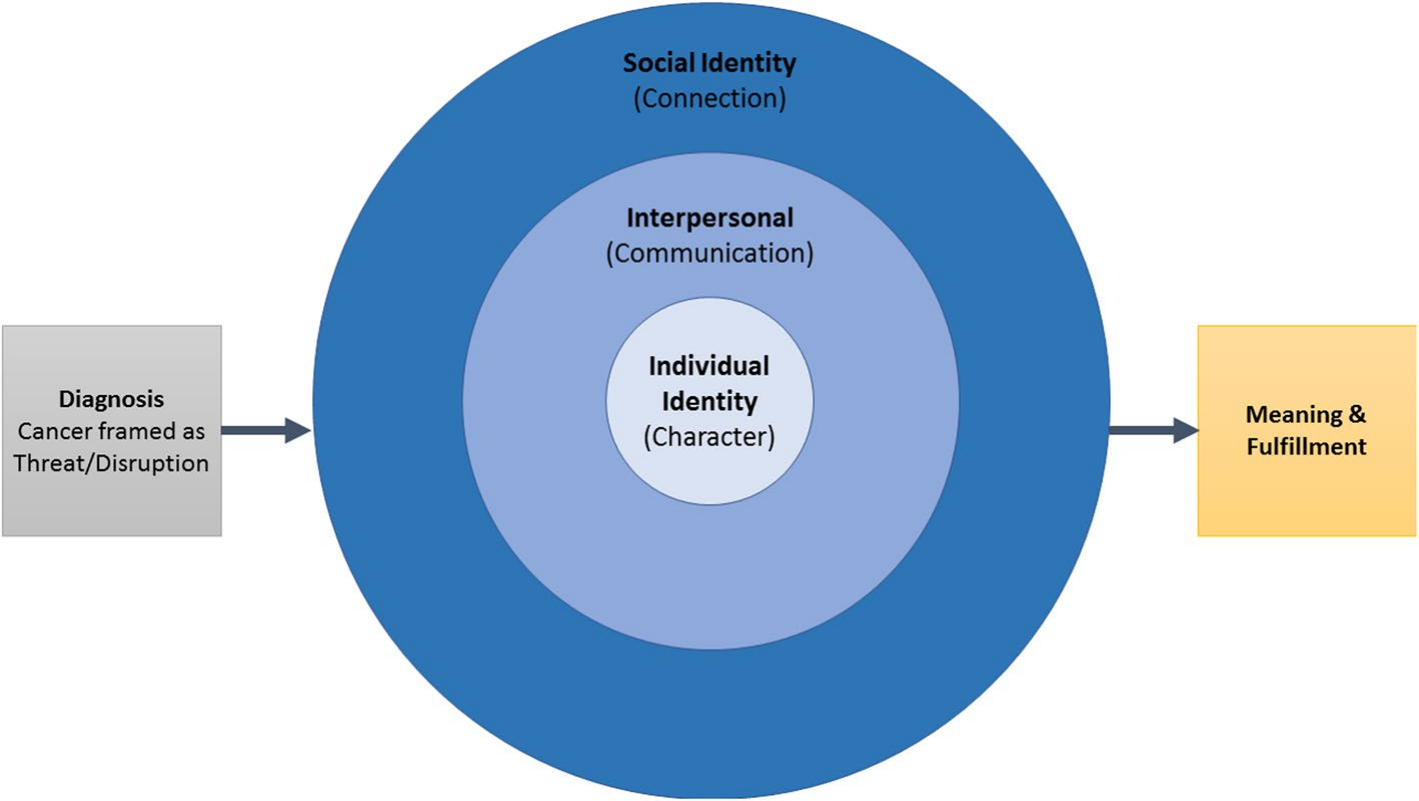
Theoretical framework: patients’ navigation of a cancer diagnosis through discussion of values

Analysis of values summaries generated 115 descriptive and interpretive codes organized into 12 categories. A detailed review of these categories (Table 2) grouped them into five thematic constructs, resulting in a theoretical framework (Fig. 1).

**Table 2:** Major Themes and Associated Codes

Thematic Construct	Category	Prominent Codes
Cancer as Threat/Disruption	Physical Impact	Concerns: Body Changes/Procedures/Diet
		Definition of Living Well: Live Without Pain/Suffering
	Treatment	Concerns: Treatment Effects
		Concerns: Treatment not Working
	Disease Trajectory & Death	Concerns: Uncertainty of Disease Trajectory
		Hopes: More Time/Live Longer
Character	Functional Independence	Concerns: Burden to Others
		Personhood: Preferences for Independence
	Maintaining Identity & Autonomy	Sources of Strength: Control
		Sources of Strength: Myself/Caring for Self
	End-of-Life Preferences/ Preferences in a Crisis	End-of-Life Preferences: Depends on Health
		End-of-Life Preferences: Dying with Dignity
Communication	Communication with Loved Ones	Concerns: For Family/Others
		Discussion with Family
	Communication with Medical Team	Personhood: Communication/Info. Preferences
		Sources of Strength: Possibility of Cure
Connection	Connection to Loved Ones	Sources Strength: Family
		“Can’t Live Without It”: Social/Emotional
	Connection to Medical Team	Sources of Strength: Medical Team
		Personhood: Relationship to Medical Team
Sources of Meaning and Fulfillment	Maintain Normalcy	Definition of Living Well: Maintain Normalcy
		Definition of Living Well: Ability to Work
	Meaning & Fulfillment	Definition of Living Well: Enjoying the Present/Living in the Moment
		Hopes: Volunteer/Help Others Like Them

These thematic constructs demonstrated patients’ focus on retaining a sense of agency, personhood and connection amidst practical and existential disruption caused by cancer. The theoretical framework depicts a pathway through which patients articulate their values, divided into five constructs beginning with the cancer diagnosis, three nesting constructs of maintaining identity and autonomy (character), interpersonal (communication) preferences and needs, and social identity (connection), resulting in identification of sources of meaning and fulfillment.

#### Construct 1: the existential disruption of cancer frames patients’ expression of their values

Several values summaries depicted cancer as a profound disruption to patients’ life “status quo” and a threat to their role in the family unit as well as to their physical and spiritual wellbeing. As one 42-year-old man with stage IV colon cancer stated, “*Being diagnosed with cancer, changes your perspective on life,*” and a 37-year-old man likened his diagnosis of stage III rectal cancer to, “*having my future stolen from me.*” This disruption was comprised of several factors, including the uncertainty of the disease trajectory (e.g., recurrence or progression) and prognosis, and the physical impacts of the cancer and its treatment. Concerns about dying commonly emerged (independent of cancer stage or diagnosis) in response to the question “Facing cancer, what concerns you the most?”, along with the desire in the face of death to either maintain or regain control over one’s life. A woman with stage III colon cancer spoke of being *“most concerned about how many years I have left and how best to spend my time.”* Worries about death from cancer often related to leaving loved ones or financial obligations behind, such as “*dying and leaving earlier than I planned. We recently got married, so we’re trying to figure out what we can do and enjoy now*” (58, M, stage IV pancreatic cancer).

#### Construct 2: expression of character (personhood) and a desire to retain a sense of self

Across all values summaries, participants voiced a desire for independence in their lives. In response to the prompt, “The following is so critical to my life that I can’t imagine living without it,” patients emphasized the importance of physical ability and autonomy in preserving a sense of self and wellbeing. Primary concerns revolved around placing burden on loved ones (*“I am most concerned about becoming debilitated, not being able to get around or do the things I need to do for my family.”* [74, M, MDS]) and being unable to fulfill roles and responsibilities within their families (*“I would never want to get to a point that [my wife] is only taking care of me. I take a lot of pride in being able to care for my family.”* [84, M, MDS]).

In the context of their individual sense of identity, patients also spoke of agency in crisis planning and communicating preferences for end-of-life care. For example, when asked to complete the prompt “in an unexpected crisis situation, I would want…” a 54-year-old man with stage IV rectal cancer stated, *“My focus is on quality of life and when the end of life comes, I want to die in a dignified way - a natural way. That is why we put the DNR order in place.”* Patient end-of-life care preferences prioritized quality of life: almost every patient rejected the notion of life-sustaining measures that would not restore a “reasonably good” quality of life. As a 67-year-old man with stage IV pancreatic cancer described, *“if there is no medical care that can get me back to some reasonable quality of life then I wouldn’t want those types of treatment.”* Patients articulated a preference to keep “fighting” only if the outcome would change, weighing the burden of life-sustaining intervention against possibilities for the future: *“I would want everything done, as long as there was a chance that I would recover with good quality life. I would not want a breathing tube or feeding tube if I would need them indefinitely”* (68, F, MDS). These end-of-life preferences were associated with the desire to preserve autonomy and avoid burdening loved ones: *“If I couldn’t eat, drink, or talk on my own I would prefer to die naturally because I’m very independent. I don’t want to suffer and put my family in more suffering; I don’t want to be on a life support machine”* (69, M, stage IV cholangiocarcinoma).

#### Construct 3: communication with loved ones and the medical team as a tool for maintaining individual agency

Layered on the individual character themes was how communication with loved ones about their treatment and values enabled participants to proactively retain their familial roles. As a 70-year-old man with MDS remarked, *“This weekend I sat with my kids and my niece and we talked about the plan moving forward, and what we understand things will look like when I go to transplant […] I want to make sure they all understand what it is going to be like.”* In addition, in response to the first question in the values conversation, “What should we know about you as a person to take best care of you?” [11] answers usually revolved around communication preferences, including communication with the medical team. Almost all patients in the sample indicated a desire for “direct” and “upfront” communication: *“I’d like for you to be completely truthful with me. I appreciate honesty and straightforwardness”* (36, M, stage IV colon cancer). For many patients, the preference for open communication became a way to assert agency and maintain a sense of control while navigating an uncertain future: *“I appreciate that you guys talk through all the options with me, let me be a partner in my care instead of just telling me what to do […] I just feel best when I know that my opinion is considered”* (64, M, MDS).

#### Construct 4: connection to others represents a core social identity and source of individual strength for patients

Patients drew strength from their family and social support system after a cancer diagnosis, and maintaining social ties was a core value during treatment. When asked “Facing cancer, what gives you strength?” a 51-year-old woman with stage III rectal cancer stated, *“I have an incredible support group of family, friends, coworkers. I’m fighting this for them because they have been unbelievably supportive.”* Many participants defined “living well” as continuing to play an active role in their family*: “It is important that I can help my son with things. If not with money, with his homework and school projects. He doesn’t tell me about girls yet, but I hope he asks me for help with dating”* (34, F, MDS). This theme was echoed in participant comments relating to their confidence and trust in their medical team, for example, *“I trust [my oncologist] and the rest of the medical team, I trust your expertise in guiding me through this”* (75, M, stage IV rectal cancer). Patients’ desire to reach key milestones with loved ones (e.g.*,* graduations, weddings, births, etc.) was referenced as a source of strength and hope; as a 72-year-old woman with MDS described, *“I want to see my grandchildren graduate, from everything I can (grammar school, high school, college).”*

#### Construct 5: sources of fulfillment and life’s meaning

Summaries showed how patients strived to draw on their Character, Communication and Connection (Fig. 1) to achieve a sense of meaning during cancer. Across all diagnoses and stages, patients desired maintenance or return to “normalcy.” When asked “What does living well mean to you at this time in your life?”, patients most often defined “living well” in terms of normalcy. According to a 64-year-old man with MDS, *“Living as close to a normal life as possible… won’t be possible right after transplant, but I hope I get through it easily as possible…”* Another patient remarked that *“I want to live the life [my partner and I] have been living and continue it for as long as possible”* (M, 67, stage IV pancreatic cancer).

Maintaining a sense of purpose was a priority for most patients, either through remaining healthy enough to work or resume pre-diagnosis responsibilities, or, serve as a source of strength or guidance for others facing a similar illness. Values summaries revealed not only what was important to patients in the context of their cancer diagnosis, but also *why* such things were important. For example, the 42-year-old man with stage IV colon cancer noted that *“Living well also means being able to spend quality time with my family members.”* In addition, the values summaries not only illustrated what was meaningful to patients, but also represented a tool for the medical team to ensure that individual care plans would honor patients’ specific values, goals and preferences.

### Differences in values between GI cancer and MDS patients

Some values spanned disease types, such as desiring detailed and frequent communication of medical details with healthcare teams, whom patients trusted and felt connected to. Patients from both groups also prioritized quality of life in establishing their preferences regarding use of life-sustaining medical treatments. These details are shown in Additional file 2, as are differences between the groups that arose in analysis.

Within Construct 1 (“Cancer as a threat/disruption”), GI cancer patients spoke more frequently about prognosis than MDS patients, who appeared more concerned about treatment choices and processes. Construct 2 (“character”) highlighted differences in the desire for independence; GI patients spoke of independence in the context of control and autonomy, while MDS patients related independence to playing an active role in their family, expressing fears of becoming a burden to their loved ones. There were no major differences in Construct 3 (“Communication”), with both groups valuing direct communication. Within Construct 4 (“Connection”) GI cancer patients mentioned their professional life as a source of strength and identity more frequently than patients with MDS, who emphasized connection to loved ones. Similarly, differences in Construct 5 (“meaning/fulfillment”) revealed a greater focus on maintaining normalcy among GI patients, while among MDS patient focused on cultivating a “new normal” in the context of their diagnosis.

## Discussion

This analysis demonstrates how values discussions allow patients to generate, process, and articulate their individual views of life’s meaning from the time of diagnosis. The resultant framework comprises a pathway: beginning with the perceived threat of a cancer diagnosis, prompting consideration of individual identity, interpersonal preferences and needs, and social identity, with convergence of these constructs to frame and support expressions of meaning and fulfillment. Patients who engaged in values discussions from the outset of cancer emphasized specific preferences, hopes, and concerns to the team from whom they receive care. Nearly universally, across two very different types of cancer, patients expressed a desire for normalcy. Thus, the values discussion revealed ways in which patients clarified their thinking about their values and provided a comprehensive look into “who the patient is”, and what matters to them, both before and after a cancer diagnosis. Thematic differences between GI and MDS patients likely reflected differences in outcome expectations and therapeutic options facing the patients in the two groups. In addition, the younger median age of GI patients may account for their greater emphasis on professional identity as a source of strength.

With detailed data available on 67 patients, this is a robust qualitative analysis of patient values in the context of a newly diagnosed cancer. This work builds on previous initiatives which have incorporated patient values, including the Living Well Interview and the Serious Illness Care Program [8, 15–19]. However, other investigations have primarily focused on what is important to patients specifically at the end of life [7, 16, 20, 21]. While some of the patients in our study echoed values seen in studies [22–24] examining end-of-life priorities (e.g., a desire to not pursue life-prolonging treatments like cardiopulmonary resuscitation or mechanical ventilation), the timing of values interviews in the present study provided a broader swath of values. We believe this early timing results in a more holistic approach to eliciting patient values, allowing for the expression of priorities both in the present (e.g., hoping for optimal cancer treatment outcomes) and in the future (e.g., not burdening loved ones should an illness result in debility unacceptable to the patient). This study adds to the evidence identified in a recent review [25] that values provide a more meaningful and durable framework the end-of-life preferences alone for decision-making throughout cancer, while affirming personhood and dignity. Thus, the themes identified in this analysis are informative for research and clinical practice early at the time of cancer diagnosis, and for all patients, both those with and without advanced disease. Other strengths of our analysis included the rigorous qualitative methodologic process, the involvement of coders from a diverse biopsychosocial training background, and the variety of patient diagnoses, both within the broad spectrum of gastrointestinal (GI) cancers, and the inclusion of participants with myelodysplastic syndrome (MDS).

Our study has limitations. While this is a relatively large qualitative patient sample with diverse disease types and cancer stages included, it was nonetheless drawn from a small number of clinics at a comprehensive cancer center. In addition, despite the large sample size, the values statements were relatively brief (one-page) and thus do not capture the detail and nuance of individual experience that may be obtained from other qualitative methods (such as in-depth interviews). The values summaries were written by nurses; therefore, some language may not be from the patients themselves despite attempts to capture quotes verbatim. However, these summaries were shown to patients, who explicitly verified them. In addition, patients and families maintain deep trust in nursing professionals [26], especially those with whom they have ongoing relationships, therefore, what patients share with these trusted clinicians may be true reflections of their personhood. While we took many steps to ensure rigor throughout our coding process (through a multidisciplinary coding team, regular consensus meetings, quality assurance checks), we did not engage patients in member checking of the final theoretical framework.

In the future, we plan to quantitatively examine how values may differ according to patients with distinct demographics (e.g., age, ethnicity), and stages (e.g.*,* late versus early), as well as how values relate to other data we collected on patients, such as their illness and treatment intent understanding, their information preferences, and symptoms. Further investigation is needed into how much patients share their values with loved ones at post-baseline, as our data reflect that most people take strength in family as a support system during cancer care. We are currently studying the feasibility and effects of Spanish-speaking cancer patients having values conversations occur with chemotherapy nurses during treatment. The framework arising from these data also provide a foundation for the development of a measure to assess and improve the myriad ways patients and families cope with cancer diagnoses. Importantly, this framework can also be used as a communication guide for clinicians as they help patients think through their values, priorities, and concerns after a cancer diagnosis.

## Conclusion

In conclusion, we successfully identified the values of newly diagnosed cancer patients and constructed a theoretical framework through which patients navigate and express their values. In an era of increasing biomedical sophistication and complexity in cancer care, patient values can and should be used by care teams as guideposts to help patients from the start and be revisited regularly as their clinical course unfolds, and in the workflow deemed most appropriate and valuable by each individual clinical team. Myriad opportunities include clinicians leveraging a patient’s stated desire for detailed communication about prognosis; responding to fears patients state about cancer treatment outcome; building optimal support systems in families and communities; and revisiting originally-stated preferences for end-of-life care as death approaches in patients with debilitating and advancing disease. Nurse-led discussion summaries make patient values accessible and visible to the entire care team, and this foundation for future goals-of-care discussions potentiates high quality palliative care, at both primary and specialty levels [27]. These values, organized within the framework identified in our analysis, can help clinicians and others understand how patients navigate the threat of their diagnosis and, ideally, envision an outcome that is individually meaningful.

## Supplementary Information

**Additional file 1.** Values Summary Template.

**Additional file 2.** Key Thematic Constructs and Illustrative Quotes.

## Data Availability

The data that support the findings of this study are available from the corresponding author upon reasonable request.

## Abbreviations

GI: Gastrointestinal
MDS: Myelodysplastic syndrome
