# Supplementary material for: Navigating a newly diagnosed cancer through clinician-facilitated discussions of health-related patient values: a qualitative analysis
Source: BMC Palliat Care. 2022 Mar 6;21:29. doi: 10.1186/s12904-022-00914-7 (PMC8898465; doi:10.1186/s12904-022-00914-7)
Supplement: Supplementary file 1 — Additional file 1. Values Summary Template. [file 12904_2022_914_MOESM1_ESM.pdf]

At recent visits, you shared with us some thoughts about what is important to you. Your nurse has summarized this discussion below. If you feel that the summary reflects your thinking, we plan to place it in your medical record, so that it is available to your full care
team here at MSK and can help guide your care. We will also provide the summary to you and encourage you to share it with your family. Lines of communication are always open between your MSK team and you. We will provide more opportunities to talk with you about your values and goals. We welcome you to continue the discussion with us, and we can revise this document whenever you would like.

About My Values

DATE OF DISCUSSION: ____________________

In order to take the best care of me, you should know:

Facing cancer, I find strength in:

I am most concerned about:

At this time in my life, living well means:

I hope for:

Other things I am hopeful for:

The following is so critical in my life I can't imagine living without it:

In an unexpected crisis situation, I would want:

I [have/have not] spoken about my values and goals with a friend, family member, or other person in my life.
