# Supplementary material for: Navigating a newly diagnosed cancer through clinician-facilitated discussions of health-related patient values: a qualitative analysis
Source: BMC Palliat Care. 2022 Mar 6;21:29. doi: 10.1186/s12904-022-00914-7 (PMC8898465; doi:10.1186/s12904-022-00914-7)
Supplement: Supplementary file 2 — Additional file 2. Key Thematic Constructs and Illustrative Quotes. [file 12904_2022_914_MOESM2_ESM.docx]

**Supplementary Appendix 2:** Key Thematic Constructs and Illustrative Quotes

| **Cancer as a Threat/Disruption** | **Illustrative Quotes:** | |
| --- | --- | --- |
| Life Disruption (*e.g.*, physically, spiritually, etc.) | - *“My plan about a year ago was to buy a condo in [Virginia] near my one daughter and grandchildren but as my health changed over this past year, I needed to be realistic. I now know this is not possible, at least not right now.” (MDS patient, F, 73)* - *“We have a cruise planned for September to Alaska, and I have a photography job in August that I would really like to be able to do. I am not sure what the plan is going to be, I think I am starting chemo soon, but I am not sure when and what it means.” (MDS patient, M, 70)* - *“Being diagnosed with cancer, changes your perspective on life.” (GI Cancer patient, M, 42)* | |
| Treatment | - *“I am concerned that we are not doing enough to treat my disease. I understand that MDS is a disease that we just monitor, but I wish there was something more active I could do.” (MDS patient, M, 72)* - *“With treatment maybe I will have a worse outcome than without the treatment.” (MDS, M, 48)* - *“The unknown, will this chemotherapy shrink the tumor, will surgery be an option. At this time, we can’t say 100% either way.” (GI Cancer patient, M, 42)* | |
| Disease Trajectory & Death | - *“Having my future stolen from me.” (GI Cancer patient, M, 37)* - *“I am most concerned about the unpredictability of things; one week could be good and the next could be bad.” (GI Cancer patient, M, 65)* - *“Dying and leaving earlier than I planned. We recently got married, so we’re trying to figure out what we can do and enjoy now, whether it be a short-term honeymoon or vacation.” (GI Cancer patient, M, 58)* | |
| Thematic differences between GI Cancer and MDS patients | **GI Cancer** | **MDS** |
|  | - Uncertainty of disease: frequent mention of time (*e.g.*, “how much do I have left?”) | - Making the right choices regarding their health/care - Concern about finding a donor |
| **Character** | **Illustrative Quotes:** | |
| Functional Independence | - *“I can't imagine living and not being able to interact with my grandkids, kids, and [my wife]. We aren't as young as we used to be, and there are some weeks that I need more help and weeks that she needs more help. We have balance. I would never want to get to a point that she is only taking care of me. I take a lot of pride in being able to care for my family. I know it sounds old fashioned or traditional, but I feel like that is my job as a man.” (MDS patient, M, 84)* - *“The experience I had with my sister's illness has informed the choices I would make if I got very sick. She became dependent on those around her and would never want that for myself.” (MDS patient, F, 69)* | |
| Maintaining Normalcy | - *“Having the energy to do the things I love: Working, being a mommy and wife. It's really about having energy and positivity. I want to enjoy life (getting my nails done, for example). With this diagnosis, I can no longer be spontaneous, because my schedule is dependent on how I feel.” (GI Cancer patient, F, 41)* - *“Regaining as much of a sense of normalcy as possible. My wife and I enjoy going to the movies and to plays, and it has been hard to do anything I enjoy because of all my appointments. Regaining independence and doing what I want, like going to [Florida] for a time, is very important to me.” (MDS patient, M, 74)* | |
| Desire to Live | - *“To get through with treatment with flying colors. Furthermore, I want to no longer rush life. I hope to go back to do things I used to love: Painting, gardening, writing, and making a family documentary.” (GI Cancer patient, F, 72)* - *“To beat this cancer, to get a chance to live a bit longer as long as possible.” (GI Cancer patient, M, 63)* | |
| End of Life | - *“If I couldn’t eat, drink, or talk on my own I would prefer to die naturally because I'm very independent. I don't want to suffer and put my family in more suffering, I don't want to be on a life support machine.” (GI Cancer patient, M, 69)* - *“I would want everything done, as long as there is a chance that I will recover with good quality life. I would not want a breathing tube or feeding tube if I would need them indefinitely.” (MDS patient, F, 69)* | |
| Thematic differences between GI Cancer and MDS patients | **GI Cancer** | **MDS** |
|  | - Independence: achieved by reversing procedures (*i.e.*, colostomy bag) and getting symptoms under control - Independence for themselves (*i.e.* feeling well enough to work or exercise) | - Independence more frequently desired for benefit of others (*i.e.* feeling well enough to take care of children) |
|  | Both GI Cancer and MDS patients want all life-sustaining measures done *only if* there was a chance of recovery with a reasonable QOL. | |
| **Communication** | **Illustrative Quotes:** | |
| Communication with Loved Ones | - *“It is important to be supportive of my family, although I understand that that role will evolve as time goes forward. I know that the emotional and social support I provide my family is as, if not more, important than the things I used to do.” (MDS patient, M, 74)* - *“Over the past few weeks, I’ve also told my friends about my cancer and they have been very supportive.” (GI Cancer patient, M, 55)* | |
| Communication with Medical Team | - *“I’d like for you to be completely truthful with me. I appreciate honesty and straightforwardness.” (GI Cancer patient, M, 36)* - *“I like lots of information. I love knowing all my numbers. I appreciate that you guys talk through all the options with me, let me be a partner in my care instead of just telling me what to do. I have been sick for several years; this isn't our first time dealing with serious illness, so I am very realistic. I just feel best when I know that my opinion is being considered.” (MDS patient, M, 64)* | |
| Thematic differences between GI Cancer and MDS patients | No major differences between the two groups; both generally appreciate direct, open communication and as much information as possible | |
| **Connection** | **Illustrative Quotes:** | |
| Connection to Loved Ones | - *“Spending time with my friends and my family. Cancer really teaches you who your friends are. It is important that I can help my son with things. If not with money, with his homework and school projects. He doesn't tell me about girls yet, but I hope he asks me for help with dating.” (MDS patient, F, 35)* - *“I rely on my family for support, brothers and cousins in Hong Kong. The birth of my twin nieces has brought me a lot of joy.” (GI Cancer patient, F, 71)* - *Spending as much quality time with my family. I also want to continue to enjoy work that has given me such pleasure. I’ve been in education for over 5 decades. A lot of my focus now is on family celebrations and trips with them. Life has been good to us and God has been good to us and I need to express my gratitude by helping my siblings who are in need.” (GI Cancer patient, F, 72)* | |
| Connection to Medical Team | - *“I follow the instructions of the medical team. I put a lot of trust in the medical team.” (GI Cancer patient, F, 77)* - *“I like to share experiences in an open and upfront way, without scaring someone. I want to be part of the team. I like responsiveness to calls, interactions are important.” (GI Cancer patient, F, 69)* | |
| Thematic differences between GI Cancer and MDS patients | **GI Cancer** | **MDS** |
|  | - More frequent mention of working/professional life as part of core social identity & source of strength - Family & medical team were key sources of strength | - Like GI Cancer patients, MDS patients found strength in connectedness to the medical team - Desire to reach key milestones with loved ones |
| **Finding Meaning/Fulfillment** | **Illustrative Quotes:** | |
| Meaning/Fulfillment | - *“Seeing my friends (I have a group of 14 close friends), so I hope to continue with our regular activities of shopping and going out to eat. Also, I want to do something meaningful with my life and help those less fortunate than me.” (GI Cancer patient, F, 71)* - *“Living as close to a normal life as possible. Which I know won't be possible right after transplant, but I hope I get through it easily as possible, I look forward to returning to some sense of normalcy. Health has been dictating my life for so long, both of our lives, I just look forward to a day that I feel more in control of things.” (MDS patient, M, 64)* | |
| Thematic differences between GI and MDS patients | **GI Cancer** | **MDS** |
|  | - Hope for normalcy - More mention of working being part of their normalcy | - Cancer reshapes planning for the future |
